# Biomechanics of sound production in high-pitched classical singing

**DOI:** 10.1038/s41598-024-62598-8

**Published:** 2024-06-07

**Authors:** Matthias Echternach, Fabian Burk, Marie Köberlein, Michael Döllinger, Michael Burdumy, Bernhard Richter, Ingo R. Titze, Coen P. H. Elemans, Christian T. Herbst

**Affiliations:** 1 Division of Phoniatrics and Pediatric Audiology, Department of Otorhinolaryngology, LMU University Hospital, Marchioninistr. 15, 81377 Munich, Germany; 2 Department of Otorhinolaryngology and Plastic Surgery, SRH Wald-Klinikum Gera, Strasse des Friedens 122, Gera, Germany; 3 Division of Phoniatrics and Pediatric Audiology, Department of Otorhinolaryngology Head and Neck Surgery, University Hospital Erlangen, Waldstr. 1, 91054 Erlangen, Germany; 4 Department of Medical Physics, Department of Radiology, Faculty of Medicine, Medical Center-University of Freiburg, Breisacher Str. 60, 79106 Freiburg, Germany; 5 Institute of Musicians’ Medicine, Freiburg University Medical Center and Faculty of Medicine Freiburg University, Elsässer Str. 2m, 79110 Freiburg, Germany; 6 Utah Center for Vocology, 240 S 1500 E, Room 206, Salt Lake City, UT 84112 USA; 7 Vocal Neuromechanics Lab, Sound Communication and Behavior Group, Department of Biology, University of Southern Denmark, Campusvej 55, DK-5230 Odense M, Denmark; 8 Department of Behavioural and Cognitive Biology, University of Vienna, Djerassiplatz 1, 1030 Vienna, Austria; 9 Janette Ogg Voice Research Center, Shenandoah Conservatory, Winchester, VA USA

**Keywords:** Biophysics, Physiology

## Abstract

Voice production of humans and most mammals is governed by the MyoElastic-AeroDynamic (MEAD) principle, where an air stream is modulated by self-sustained vocal fold oscillation to generate audible air pressure fluctuations. An alternative mechanism is found in ultrasonic vocalizations of rodents, which are established by an aeroacoustic (AA) phenomenon without vibration of laryngeal tissue. Previously, some authors argued that high-pitched human vocalization is also produced by the AA principle. Here, we investigate the so-called “whistle register” voice production in nine professional female operatic sopranos singing a scale from C6 (≈ 1047 Hz) to G6 (≈ 1568 Hz). Super-high-speed videolaryngoscopy revealed vocal fold collision in all participants, with closed quotients from 30 to 73%. Computational modeling showed that the biomechanical requirements to produce such high-pitched voice would be an increased contraction of the cricothyroid muscle, vocal fold strain of about 50%, and high subglottal pressure. Our data suggest that high-pitched operatic soprano singing uses the MEAD mechanism. Consequently, the commonly used term “whistle register” does not reflect the physical principle of a whistle with regard to voice generation in high pitched classical singing.

## Introduction

Operatic solo singing requires electronically un-amplified vocal sound production at sound levels that are suitable to compete with large orchestras and choirs. This kind of voice production for artistic purposes extends the fundamental frequency (*f*_*o*_) range well beyond what is used in human speech communication, where the average *f*_*o*_ is at about 120 Hz and 200 Hz for adult males and females, respectively^1^. The entire singing *f*_*o*_ range can only be covered if the different laryngeal production mechanisms available to the human voice—often termed voice “registers”^2,3^– are utilized.

The two main laryngeal mechanisms are mechanism **M1** (also frequently termed the “chest” or “modal” register, and typically used in speech and often in singing) and **M2** (also frequently termed “falsetto” or “head” register, mainly used in singing, but sometimes also in speech). The most extreme upper musical pitch range of operatic sopranos—typically sung by adult human females, who are generally known to phonate at higher frequencies than males due to their shorter vocal fold length—extends the voice to an *f*_*o*_ range of about 1000–1600 Hz, or about three octaves above the *f*_*o*_ of speech. This range, which is regularly accessible to professionally trained classical soprano singers, is acoustically characterized by a strong fundamental and weak overtones in comparison to other laryngeal mechanisms^4,5^, justifying a classification into a separate mechanism, M3. This **M3** voice production mechanism is commonly being called the “whistle register” (German: *Pfeifstimme*—cf.^6^) in singing voice pedagogy^7^. There is no clear definition of the actual range and mechanism of that register: Garnier et al.^8^ found a transition to the whistle register already between the pitches D#5 and D6, while some authors only speak about a transition at ca. C6, and Titze would only call it whistle register from and above F6^9^. Mechanism M3 is hypothesized to be distinguishable from M2 due to differences (1) in the voice source, i. e., the laryngeal mechanism, (2) in the resonances or tuning strategy, (3) of interactions of the voice source and the resonances. Detailed essays on this can be found elsewhere^2,3,10,11^. The utilized terminology would suggest an aeroacoustic production mechanism, where a rigid structure combined with certain resonance causes airflow instabilities that produce such high frequencies.

**Figure 1:**
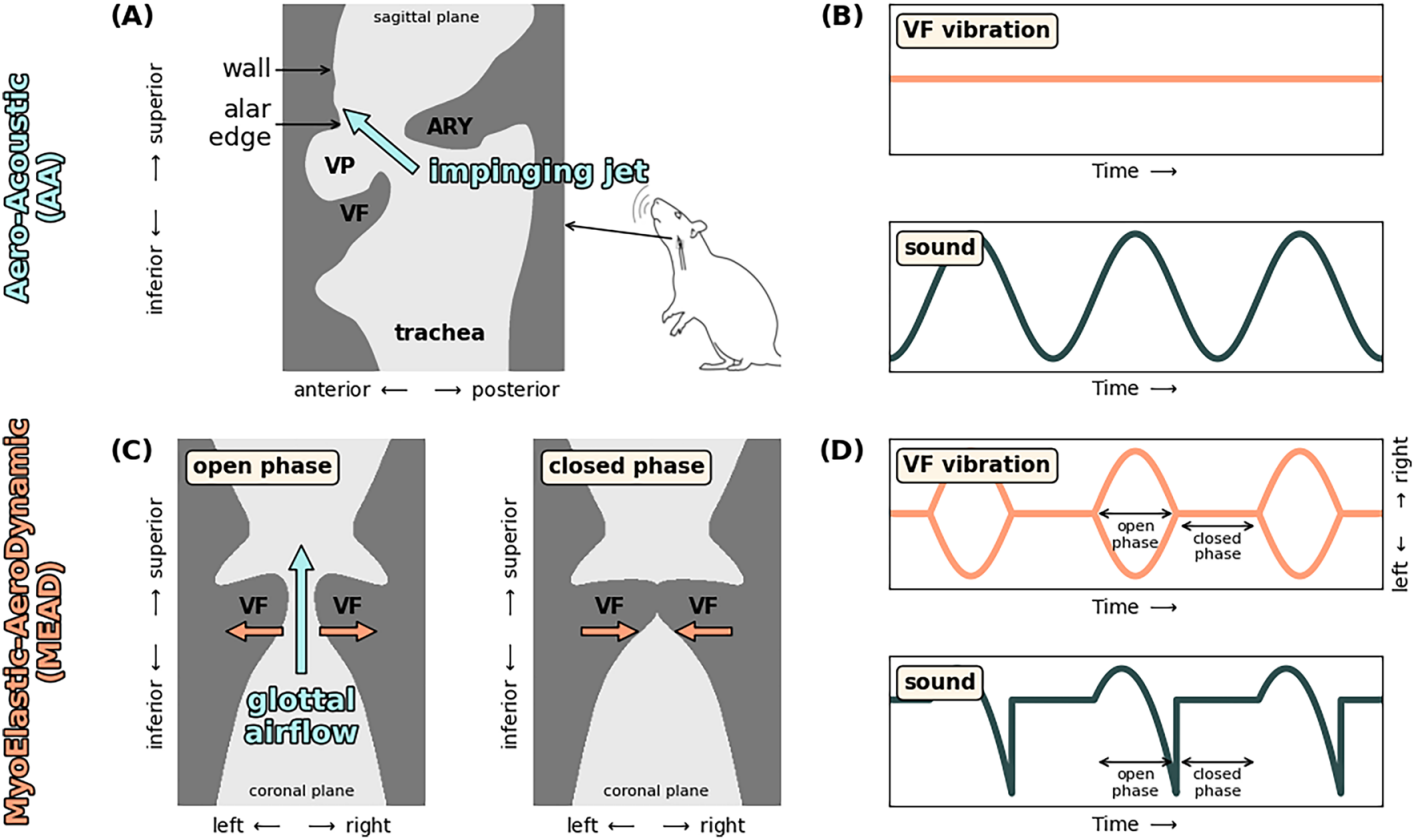
Schematic overview of Aero-Acoustic (AA) and MyoElastic-AeroDynamic (MEAD) voice production mechanisms. (**A**) Mid-sagittal view of rodent larynx, illustrating the formation of the impinging jet in AA sound production; (**B**) schematic display of the lack of tissue vibration in the AA mechanism and the resulting sinusoidal sound source; (**C**) coronal view of human larynx, illustrating the open and the closed phase of medio-lateral vocal fold vibration; (**D**) vocal fold displacement pattern and resulting prototypical acoustic voice source^22^ for the MEAD mechanism.

In contrast, humans typically produce voice through the MyoElastic-AeroDynamic (MEAD) principle^12–14^. There, the vocal folds enter a state of self-sustained oscillation. The ensuing medio-lateral oscillation of the vocal folds, successively facilitating partial or full closure of the laryngeal airway, causes cyclic modulation of the exhalatory airflow. Those airflow fluctuations translate to pressure variations which are the main constituent of the generated vocal sound^15,16^ (see Fig. 1C,D). In particular, the main acoustic excitation event is set up at the moment of airflow cessation during each cycle^17^, when the vocal folds are maximally approximated in their membranous part, often resulting in full vocal fold collision. The corresponding medio-lateral vibratory mode, resulting in air flow modulation variation, is a crucial requirement for the MEAD mechanism and thus distinguishes it from an aeroacoustic production principle.

MEAD is the predominant mechanism of sound production in mammals, extending across a range of body sizes and *f*_*o*_, spanning more than four orders of magnitude from 10 Hz to 120 kHz. However, the murine rodents—which with 1400 species comprise about 25% of all 5400 mammal species—have adapted a completely different physical mechanism of sound production to extend their high frequency vocalization range. Murine rodents—including mice and rats—produce ultrasonic vocalizations (USV) with an aeroacoustic mechanism^18^. Several aeroacoustic mechanisms have been proposed to explain USVs in rodents that differ in their local flow conditions and acoustic feedback properties: (a) wall-impinging jets^19,20^ (i.e., focused airflow that strikes an opposing surface—see Fig. 1A,B); (b) edge impinging jets (resulting from successive oscillation of an airflow jet to alternate sides of a ridge that is struck by the airflow)^21^ and (c) cavity whistles (generated through air vortex oscillation within the cavity)^20^. In laboratory rats and mice, the wall-impinging whistle drives USV production, but alternative mechanisms may be found in the large number of rodent species^19,20^. Interestingly, in these species, the respective USV production mechanism co-exists with the “conventional” MEAD mechanism, the latter being exclusively used for vocalization at lower and thus humanly audible *f*_*o*_.

Given the highly conserved laryngeal anatomy across mammals^23^, it might be possible that the operatic sopranos vocalizations at very high *f*_*o*_ are also produced by a special aeroacoustic mechanism. In point of fact, a number of authors describe the general possibility for an alternative aeroacoustic sound production mechanism in humans. This was hypothesized to be achieved by means of “chink tones” analogous to whistling^24^ in the larynx and subsequent cavity resonance”^12^, or vortex-induced vibration of the folds^25–28^, possibly involving interactions between the voice source and the vocal tract^29,30^. Such mechanisms are sometimes referenced to as **M4** or “glottal whistle” for the *f*_*o*_ range of 1–3 kHz and above^31^. In such an aeroacoustic sound production mechanism it could be expected that the frequency of the whistle *f*_m1_ becomes *f*_*o*_. Other studies showed that high-pitched human vocalization can be produced with the MEAD mechanism^5,6,8,28,32–35^. However, all those studies come with certain limitations: They either (a) had a limited number of participants for laryngoscopic examination, i. e., n = 1^8,32–34^, n = 2^35^; n = unknown^28^; (b) employed a limited data acquisition methodology which was either indirect, using electroglottography^5^, or did not allow direct observation of the vocal folds along their entire antero-posterior length^32^; and/or (c) had a limited temporal resolution (with the Nyquist frequency below *f*_*o*_), thus resulting in aliasing and preventing adequate time-resolved within-cycle documentation of the sound production mechanism^6,8,34^. Even though there is evidence for both principles, it remains uncertain which sound production mechanism and configuration are responsible for operatic singing in a range of *f*_*o*_ that resides at the bottom of the so-called whistle register range, which typically exceeds fundamental frequencies of 1000 Hz. In addition, a confusion regarding the terminology and the associated mechanisms and application in different music genres remains widespread up until today. In this study, we address the issue by providing the first comprehensive documentation of high-pitched soprano singing with super-HSV at 20,000 fps, investigating a larger cohort of professional operatic sopranos.

## Results

**Figure 2:**
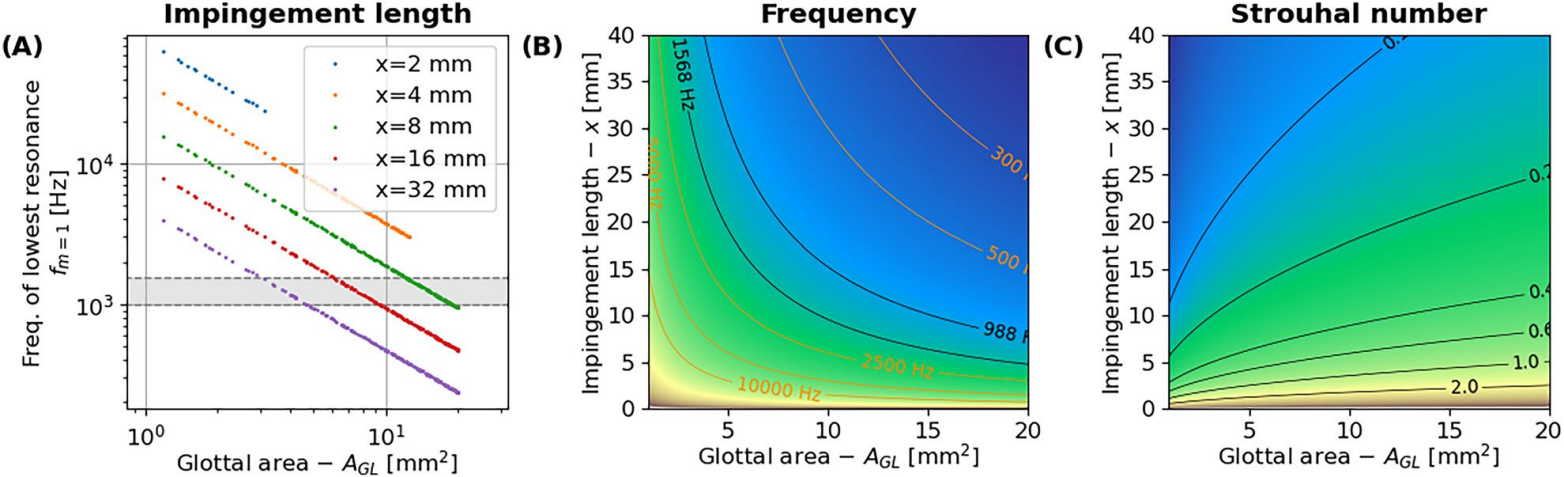
Simulation of hypothetical frequencies for impinging jet sound production in humans, using the model presented in^20^. The resulting frequencies scale linearly as a function of volumetric airflow *V*. The figure illustrates the case of *V* = 150 ml/sec, i. e., a default flow value seen in human singing^36^. (**A**) and (**B**) isoparametric curves for emerging mode-1 frequencies (i.e., lowest possible stable frequency of a whistle) as a function of glottal area (*A*_*GL*_) and impingement length (*x*). The gray area in (**A**) depicts the fundamental frequency region of the “whistle” register in female operatic singing^37^. (**C**) Strouhal number *St* (a dimensionless quantity describing the oscillatory flow mechanism). Stable vortex whistles are expected at *d/x* < *St* < 1, where *d* is the jet diameter.

First, we tested whether the wall-impinging jet model developed for rodents^20^ also applies to human voice production anatomy and physiology. Notably, our data (Fig. 2) suggest that such an aeroacoustic mechanism is hypothetically possible for high-pitched female operatic singing. The three independent parameters of that model—i. e., glottal area, impingement length (the length of the airflow jet that strikes the opposing surface), and volumetric airflow—could be gradually controlled by singers through glottal adduction, epiglottis tilt (i.e., a backwards rocking motion of the epiglottis, reducing the volume of the space just above the vocal folds), laryngeal constriction, and muscular adaptations of the pulmonary apparatus, thus theoretically allowing for gradual control of the emerging phonatory frequency in artistic contexts.

**Figure 3:**
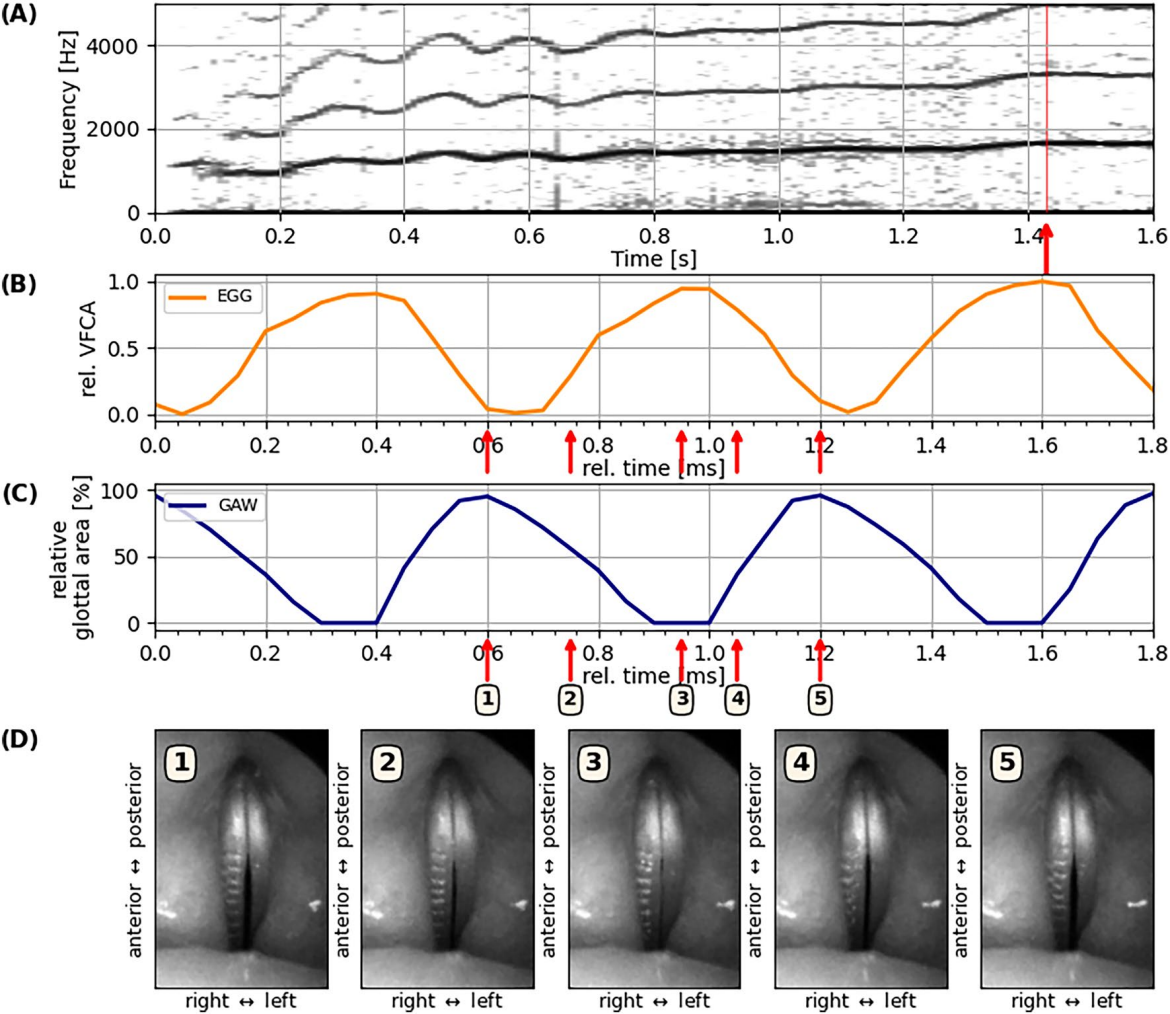
Example of high-pitched phonation of S3. (**A**) Acoustic spectrogram; (**B**) three cycles of laryngeal oscillation at *t *≈ 1.43 s, showing vocal fold contact area documented with electroglottography (EGG); (**C**) time-varying glottal area waveform (GAW), as documented by high-speed video (HSV) recording at 20,000 frames per second. The arrows indicate the still HSV frames shown in panel d; (**D**) HSV frames extracted at the incidents indicated by the arrows in panels B and C. Note the full glottal closure in the third out of the five displayed video frames.

Contrary to these results from the simple aeroacoustic model, empirical data from the nine investigated professional singers strongly suggest that the aeroacoustic mechanism is not the origin of high-pitched soprano singing. As compelling supporting evidence for the MEAD mechanism, we found vocal fold vibration and collision in all nine participants (see supplementary materials for HSV samples from all participants). In all investigated sopranos, the vibratory frequency of the medio-lateral vocal fold oscillation corresponded to the *f*_*o*_ of the radiated sound (see Suppl. Fig. S1). This suggests that the tissue oscillation is causal to sound generation, which is highly indicative of the MEAD principle. This phenomenon is documented exemplarily in Fig. 3: The HSV still images in Fig. 3D document full glottal closure along the entire visible vocal fold length. The electroglottographic (EGG) signal (panel C) shows clear cyclic variation of vocal fold contact along the sagittal glottal plane. The glottal area, i. e., the opening between the left and right vocal folds co-varied in synchrony with the EGG signal. The resulting glottal area waveform (panel B) reached a maximum when the relative vocal fold contact area, as retrieved by the EGG signal, was at a minimum. This is in good agreement with prototypical human voice production in the modal register (e. g. in speech), where the vocal fold contact assumes a maximum when the glottis is closed), and vice-versa^38^. Without exception, the acoustic signals captured from the singers resembled a harmonic series with a number of noteworthy harmonics (between two and seven) throughout the examined vocal range, from pitch C6 (*f*_*o *_*≈ *1047 Hz) to G6 (*f*_*o *_*≈ *1568 Hz) in all investigated sopranos, with participant S3 achieving phonation at musical pitch B6 (*f*_*o *_*≈ *1975 Hz—see Suppl. Fig. S2, out of the regular experimental protocol). The sound level differences (H/-H2) between the first and second harmonic of the radiated acoustic signal were in the range of 23.47 (± 8.21) dB for the lowest *f*_*o*_ and 17.20 (± 4.48) dB for the highest investigated *f*_*o*_ of each participant (see Suppl. Fig. S3,S4 for details).

**Figure 4:**
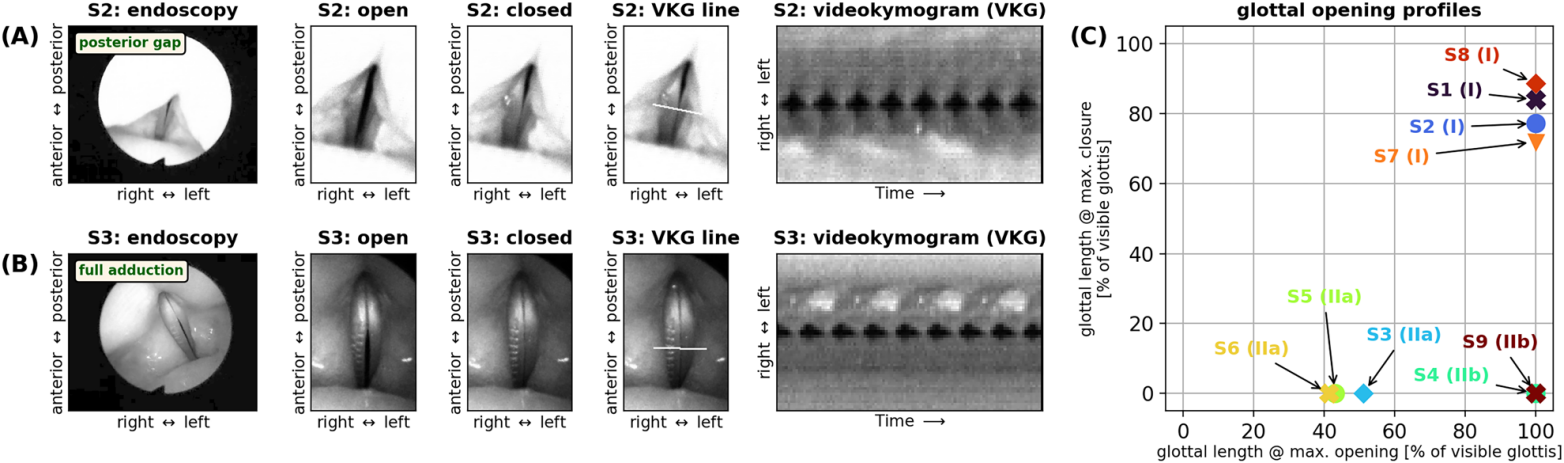
Stereotypical glottal configurations in high-pitched operatic soprano singing. Two main strategies emerged: (**A**) **glottal configuration I**: phonation with incomplete vocal fold adduction, resulting in a posterior glottal gap during vibration (even in the “closed” phase); and (**B**) **glottal configuration II**: greatly increased adduction of the arytenoids, supported by medialization of the ventricular folds; (**C**) glottal opening profiles for all strategies—see supplementary materials S5 and S6 for details.

In contrast to the “default” laryngeal configuration in classical singing, which requires a moderately low vertical laryngeal position, phonation in the high-pitched soprano range was invariably facilitated by a raised larynx and moderate to extreme medialization of the ventricular folds (see Suppl. Fig. S5,S6). This suggests that the respective *f*_*o*_ could only be achieved with pronounced larynx elevation and/or constriction. Overall, we found the following stereotypical glottal configurations at the highest examined pitches, which are documented in Fig. 4 and Suppl. Fig. S5:

- Four participants (S1, S2, S7, and S8) phonated with a posterior glottal gap (denoted as **glottal configuration I** throughout the remainder of this manuscript), suggesting incomplete vocal fold adduction (see Fig. 4A for an example). They had completely separated vocal folds (along the entire visible anterior-posterior length) in the open phase and a partially closed glottis in the closed phase (i.e., the duration of the oscillatory cycle where the vocal folds are in contact, temporarily stopping or at least greatly reducing the laryngeal air flow), with vocal fold contact along 44 % to 75 % of the visible glottal length.
- In contrast, the five other participants (S3, S4, S5, S6, and S9) phonated with full glottal closure (100 % vocal fold contact) in the closed phase, but with different configurations during the open phase. Three participants (S3, S5, and S6) had only a partial opening of the vocal folds, occurring along 40 % to 50 % of the visible vocal fold length (**glottal configuration IIa**)—see Fig. 4B for an example. The other two participants (S4 and S9) phonated with a fully opened visible glottis in the open phase (**glottal configuration IIb**).

**Figure 5:**
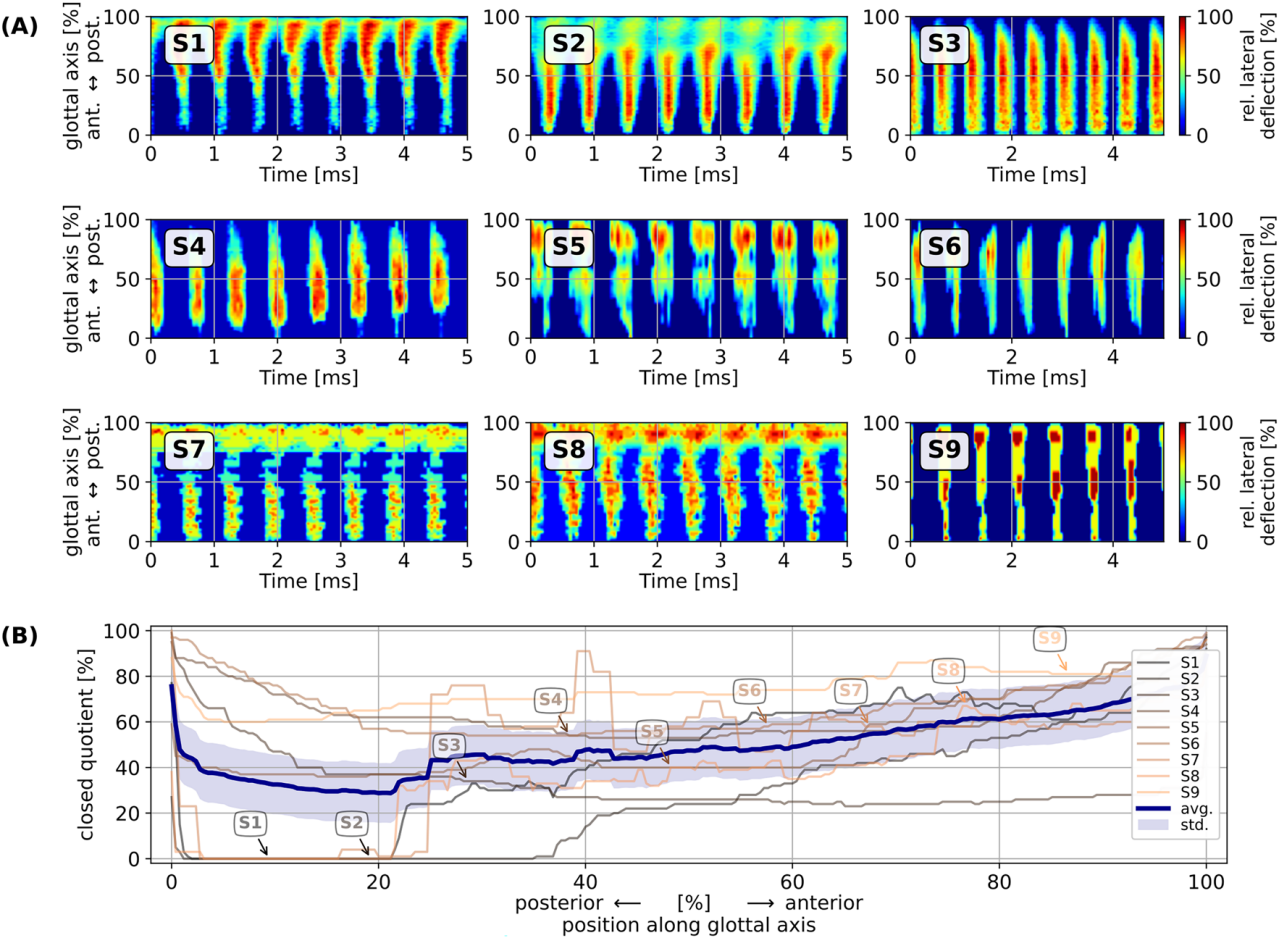
Vocal fold vibration analysis for phonation at pitch G6 (ca. 1568 Hz). (**A**) Glottovibrograms (GVG) for all investigated sopranos (S1 through S9); (**B**) individual and averaged glottal closed quotients along the antero-posterior axis (cf. Figure 4 in^39^).

Respective documentation for all sopranos is provided in the supplementary materials (Suppl. Fig. S5,S6) and in Fig. 5. These data clearly corroborate the observation made in the HSV footage: The blue areas in the detail panels of Fig. 5 for S1 through S9 (A) are indicative of glottal closure and vocal fold collision, which occurred either partially (S1, S2, S7, and S8) or along the entire antero-posterior length of the visible glottis (S3, S4, S5, S6, and S9). Due to the observed vocal fold collision, the closed quotient (CQ), i. e., the relative duration of glottal closure over one vibratory cycle, was non-zero in most instances. Averaging all computed CQ values across the entire antero-posterior glottal length across all nine participants resulted in a median CQ value of 47.6%, with 5 and 95 percentiles at 29.6% and 73.0%, respectively.

**Figure 6:**
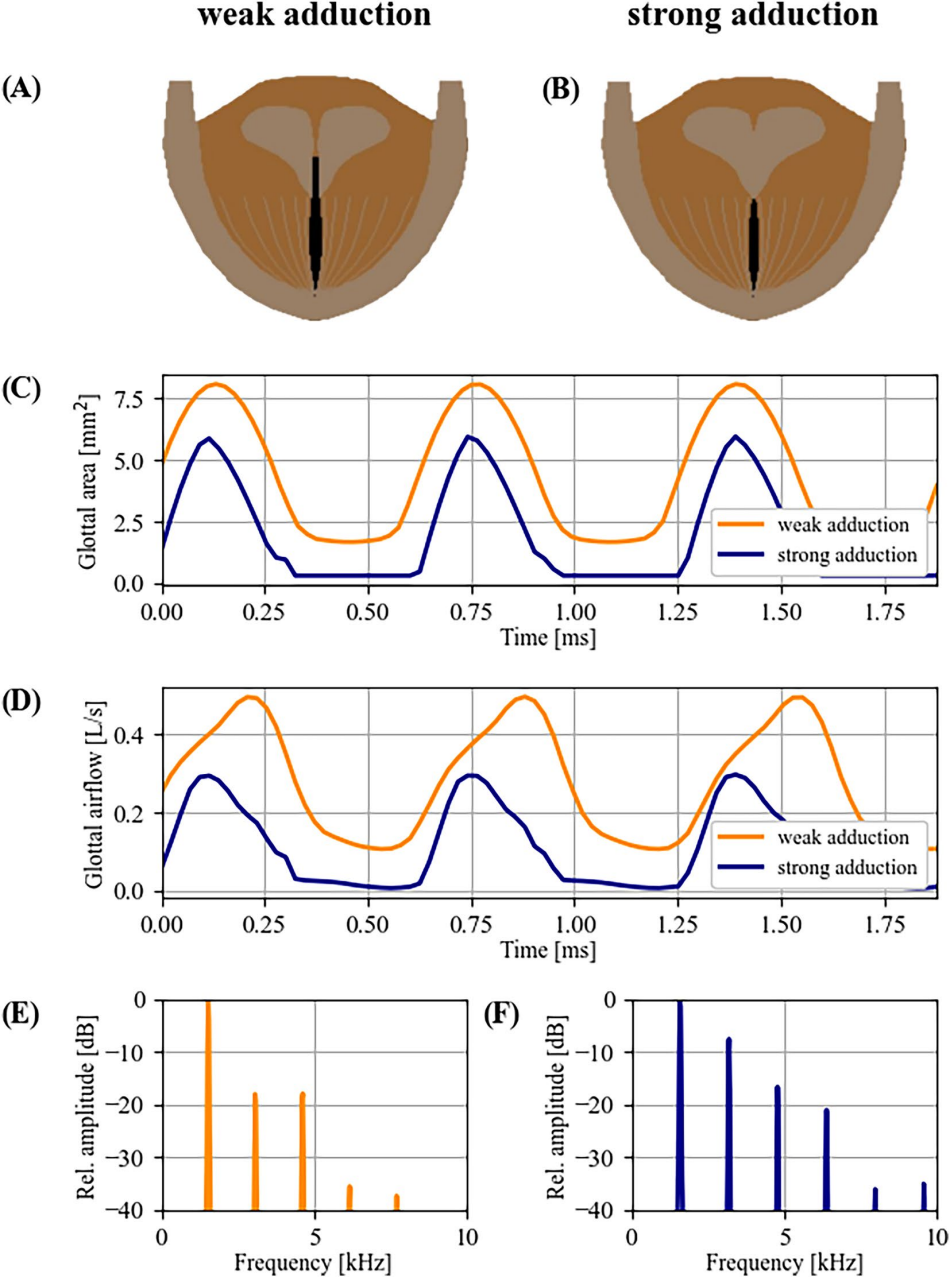
Computer simulation with a low-order finite difference model of vocal fold tissue vibration. (**A**) and (**B**) two pre-phonatory glottal configurations, resembling strong and weak adduction; (**C**) and (**D**) resulting glottal area and glottal airflow for high-pitched simulations with both pre-phonatory glottal configurations; (**E**) and (**F**) normalized spectra of glottal airflow resulting from strong and weak adduction.

We successfully reproduced the high-pitched soprano voice production with a finite difference model of vocal fold tissue vibration with string-like restoring forces. The two glottal configurations I and II were simulated with “weak” and “tight” vocal fold adduction, regulated via the pre-phonatory distance between the vocal processes of the arytenoid cartilages (with *d* = 0.6 mm and *d* = 0.1 mm for weak and tight adduction, respectively). Phonation with weak adduction resulted in a posterior glottal gap (Fig. 6A,B), all other parameters being equal across the two conditions. The emerging *f*_*o*_ was 1,540 Hz and 1597 Hz for weak and tight adduction. Results showed that string-like vocal tissue layers (mucosa and ligament), both with a fiber stress of 0.9 MPa, produced self-sustained vocal fold oscillation, again corroborating the MEAD production mechanism. This was the case for both weak and tight adduction scenarios. With weak adduction, the larger time-varying glottal area (Fig. 6C) caused larger airflow rates that were non-zero in the “closed” phase (Fig. 6D). This resulted in a reduced strength of the second harmonic in the frequency spectrum (Fig. 6F), as compared to phonation with strong adduction (Fig. 6E).

## Discussion

This study investigates high-pitched operatic phonation in the so-called whistle register above C6 using super high-speed laryngoscopy and computational modeling. Our data suggest that high-pitched soprano operatic singing is not produced by an aerodynamic whistle. Rather, we found medio-lateral vocal fold vibration synchronous to the variation of the radiated acoustic pressure in all investigated sopranos when phonating at *f*_*o *_*≈ *1.6 kHz. The observed medio-lateral vibratory component—in five out of nine singers resulting in full glottal closure—is a fundamental requirement for voice production according to the MEAD principle^15^. This medio-lateral vibratory component, resulting in a cyclical variation of the glottal area at the observed fundamental frequencies would be clearly detrimental to sound production with an aeroacoustic phenomenon. This is because a time-varying glottal area—causing time-varying airflow rates at the rate of the fundamental frequency—would introduce a considerable amount of frequency modulation (FM) into the putatively emerging aeroacoustic sound, thus violating the requirement to produce voice at quasi-stationary *f*_*o*_ conditions in artistic singing. Furthermore, the acoustic signals contained a well-defined harmonic structure, with the second harmonic having a sound level that is only about 20 dB lower than that of the fundamental. This clearly contrasts true aeroacoustic sound production like rodent ultrasonic vocalization^40^ or human lip whistling^41^, where the second harmonic’s level is 40 dB or more below that of the fundamental.

For these reasons, an aeroacoustic sound production phenomenon can be clearly ruled out for the investigated high-frequency operatic singing style. Consequently and in agreement to previous studies^5,6,8,28,32–35^, the frequently used term “whistle register”—while potentially applicable to other types of ultra-high-pitched voice production—does not reflect the physiologic voice production mechanism for classical/operatic singing at these high frequencies. Further, register names are commonly deducted rather from perceptive factors, such as “head register” and “chest register”, which are not necessarily scientifically appropriate but still established. However, it is precisely when using the term “whistle register” for the pitch range analyzed in the presented study that the underlying physiological principles should be correctly classified.

Female speech occurs at an average *f*_*o*_ of approximately 200 Hz^1^. The lowest pitches of adult females are found at about 135 Hz^1^, and the highest pitches of operatic soprano singing, investigated here, occur at about 1.57 kHz (and ca. 2 kHz in one case, see Suppl. Fig. S2), thus covering a range of almost four octaves. This is in good agreement with the predicted *f*_*o*_ range for different mammalian species at large^42^.

It is remarkable that the observed vibratory characteristics of the vocal folds documented here (recall Fig. 5B, showing median CQ values of 47.6%) closely resemble those reported for singing voice production in the M2 (“falsetto”) register found at relatively lower *f*_*o*_. For M2 phonation, Henrich et al.^43^ documented EGG contact quotients in the range of 5–50%, Herbst et al.^44^ reported videokymographic closed quotients in the range of zero to about 50%, and Echternach et al.^45 ^reported high speed videolaryngoscopically derived closed quotients of the glottal area from 0 to 50%.

Furthermore, during the review process of the presented manuscript, Kato et al. published a quite comparable study in non-professional-singers subjects, analyzing pitches from C6 to A6, however using rigid transoral laryngoscopy during high speed recordings^46^. Although a transoral rigid endoscopy might have affected vocal fold tensions and vocal tract/voice source interactions, also these authors documented vocal fold oscillations in all of their 6 subjects.

Considering these findings, we conclude that the laryngeal vibratory phenomena of high-pitched operatic soprano singing are comparable to what is seen in the M2 mechanism. The auditory perceptual distinction of the investigated type of voice production (commonly termed the M3 mechanism) from the lower-pitched M2 vocal register is likely caused by influences of the vocal tract, as suggested by previous research^47–50^.

### Types of vocal fold closure

Five of the nine participants phonated with a fully adducted posterior glottis, and in three of these the vocal folds were partially in contact along the antero-posterior mid-line at the moment of maximum glottal opening. This might be indicative of a “damping” phenomenon in analogy to violin playing—shortening the vibrating portion of a violin string with finger pressure—as previously proposed by some authors^51–54^. In such a “damping” mechanism, control would be facilitated by adjustment (and, specifically, shortening) of the vocal fold portion that is in vibration, brought about by high degrees of vocal fold adduction and arytenoid compression. It is, however, unlikely that a medial (adductory) pressure can establish a fixed boundary without excessively constricting the entire glottis. Further, the “pinning” force would be in the wrong direction. It is not perpendicular to the vibration, as in pinning a violin string, but rather in the direction of motion. This would establish a “fuzzy” boundary point, unlikely to be controllable over a wide pitch range.

The damping concept was also not supported by our computational model. For a “damping” phenomenon to occur, the physical boundaries of vocal fold vibration would have to be varied in antero-posterior direction, which was not observed in vivo and could also not be reproduced in silico. Furthermore, contraction of the thyroarytenoid (TA) muscle, which may normally contribute to medial compression during voice production in M1 and M2^2^, does not have much effect when counteracted by extremely high ligament stiffness. We therefore propose the following alternative hypothesis: MEAD phonation at the investigated range requires unusually high activity in the cricothyroid (CT) muscle in order to influence the ligament stiffness that is required for the targeted *f*_*o*_. However, all else being equal*,* such a maneuver may lead to abduction of the posterior glottis^55^, which is detrimental to voice source strength (recall Fig. 6E). Consequently, singers try to counteract this tendency to vocal fold abduction by increasing glottal closure through substantial degrees of laryngeal medialization (i. e., adduction of arytenoids, vocal folds, and ventricular folds—recall Suppl. Fig. S5,S6), in order to maximize the achievable degrees of glottal closure and thus increase the amplitudes of the higher harmonics of the voice source. The observed glottal configurations (I, IIa, and IIb) were thus most likely the result of individual anatomical and physiological predisposition.

### Findings in relation to other types of high-pitched singing

Summarizing, our investigation was concerned with investigation of high-pitched operatic soprano singing. However, ultra-high-pitched singing of both female and male singers is also found in other singing styles. Specifically, some contemporary commercial music (CCM) singers regularly phonate in the *f*_*o*_ range of 2–3 kHz^47^. Furthermore, performers singing in the “extranormal voice” style have been reported to phonate at *f*_*o*_ of up to 6 kHz^31^, and it was speculated that this “M4” phonation would be produced with a “vortex whistle”^56^. Notably in this context, Tsai et al.^26^ suggested a diffuser jet with periodic vorticity bursts in the larynx for phonation at *f*_*o *_*≈ *4 kHz. That respective phonation was investigated with ultrasound Doppler imaging, revealing a vocal fold vibratory amplitude of 0.1 mm, i.e., only barely visible to the naked eye. This is in agreement with Di Corcia et al.^28^, who reported that their participants’ “stop closure whistle” was produced during total absence of a mucosal wave and thus vocal fold vibration. These findings suggest that further investigation of the ultra-high-pitched phonations of humans is required. While—based on our data and findings—we can conclusively show that high-pitched operatic soprano singing is produced according to the MEAD principle, we cannot altogether rule out an aeroacoustic production mechanism for other human singing styles, for example CCM, contemporary experimental music, non-professional classical singing, or folk styles, at higher fundamental frequencies. In this respect, in contrast to western classically trained singing, in CCM, amplification techniques are commonly used for high-pitched singing. If an aeroacoustic mechanism would be present, it could be expected that the radiated sound would be rather weak. However, this could be counteracted by amplification. If an aeroacoustic production mechanism could be empirically documented in non-classical high-pitched singing or any other human voice production, it would be of interest to explore in future experiments if it is possible to drive the vocal folds as a passive, coupled resonator with such a sound generating mechanism.

### Limitations

The transnasal endoscopic data acquisition might have caused some irritation for the participants and it cannot be excluded that it led to tightening reflexes or increased muscle tension. However, none of the participants reported such irritation and none of them canceled the ongoing procedure. The advantage over exclusively electroglottographic measurements is the detailed observation of the vocal folds’ configuration and movement. In addition, for very high pitches, the larynx is often raised and the vocal tract tube is narrowed. This might cause slipping of the electrodes and corruption of the signal. It could be of interest how singers estimate their voice production at such high pitches, i.e. if they use AA or MEAD and if there is a full closure for the MEAD. It is a limitation of the present study that no systematic evaluation of the participants’ proprioceptive feedback concerning register transitions and phonation mechanism was performed. Such systematization of proprioception linked to actual physiology could, however, be a subject of future research.

Finally, the acquired voice signals contained aliasing artifacts above 4 kHz. However, given that the investigated fundamental frequencies were typically well below 2000 kHz, it was safe to compute both the *f*_*o*_ and H1-H2 metrics of those signals (see Methods for an in-depth discussion).

## Materials and methods

All methods were carried out in accordance with relevant guidelines and regulations. Furthermore, all experimental protocols were approved by the Freiburg University Ethical Committee (Nr 380/12). Informed consent was obtained from all subjects.

### Participants and phonatory task

Following approval from the local ethical committee, nine female professional operatic soprano singers were investigated. All participants had at least 4 years of professional training in western classical singing. Limited biographic data and a taxonomic classification according to the scheme proposed by Bunch and Chapman^57^ are provided in Table 1. At the time of the experiment none of the participants complained of any vocal symptoms, and vocal pathologies were excluded based on videostroboscopy and/or high-speed digital imaging evidence.

**Table 1:** Overview of participants, indicating their proficiency level (taxonomy according to Bunch and Chapman^57^), investigated phonatory *f*_*o*_, and observed glottal configuration.

Subject	Age	Taxonomy (Bunch–Chapman)	Highest pitch sung in study	Glottal configuration	Glottal configuration (detail)
S1	21	7.2	G6 (1568 Hz)	I	intercartilaginous posterior glottal gap (PGG), potentially reaching into membranous vocal fold portion
S2	29	7.1/4.1	G6 (1568 Hz)	I	PGG reaching into membranous vocal fold portion
S3	25	7.2	(B6 (1975 Hz) beside the protocol)	IIa	complete glottal closure, arytenoids and vocal processes pressed together, visible partial vocal fold contact along antero-posterior axis even in open phase
S4	27	7.2	G6 (1568 Hz)	IIb	complete glottal closure, arytenoids and vocal processes pressed together (but most posterior glottis not visible)
S5	36	3.1a, 4.5	G6 (1568 Hz)	IIa	complete glottal closure, arytenoids and vocal processes pressed together, visible partial vocal fold contact along antero-posterior axis even in open phase
S6	30	4.1/4.5	F6 (1397 Hz)	IIa	complete glottal closure, arytenoids and vocal processes pressed together, visible partial vocal fold contact along antero-posterior axis even in open phase
S7	31	3.1a, 3.1c	G6 (1568 Hz)	I	PGG, potentially reaching into membranous vocal fold portion
S8	46	3.1a, 3.1c	G6 (1568 Hz)	I	PGG
S9	23	7.2	F6 (1568 Hz)	IIb	complete glottal closure, arytenoids and vocal processes pressed together

The participants were asked to sing an ascending major scale on the vowel [a:] from musical pitch C6 (ca. 1047 Hz) to G6 (ca. 1568 Hz), avoiding extensive vibrato. Each note should last approximately one second. As pointed out in the introduction, there is no consensus at which musical pitch the so-called whistle register would start. However, there seems to be a general agreement that the musical pitch G6 falls within the whistle register. This motivated the choice of the pitch range in the presented study. The vowel [a:] was chosen because it could be expected that above *f*_*o*_ of 700–800 Hz classical singers will only produce this vowel quality: When *f*_*o*_ reaches the center frequency of the lowest vocal tract resonance (*f*_*R1*_), singers tend to avoid a crossing of *f*_*o*_ and *f*_*R1*_, thus raising *f*_*R1*_ as *f*_*o*_ further increases^50,58^. All except two participants (S6 and S9) successfully accomplished the phonatory task, while S6 and S9 could only reach the musical pitch F6 (*f*_*o *_*≈ *1397 Hz).

### Data acquisition

Analogous to previous investigations^45,59–61^, High Speed Digital Videolaryngoscopy (HSV) was performed using trans-nasal endoscopy. A flexible endoscope (ENF GP, Fa. Olympus, Hamburg, Germany) was mounted on a 38 mm C-mount adapter (Karl Storz, Tuttlingen, Germany), connected to a Photron high-speed camera (Fastcam SA-X2, Photron, Tokyo, Japan) and a 300 W light source (Karl Storz, Tuttlingen, Germany) which was operated at a frame rate of 20,000 frames per second (fps) and a spatial resolution of 386 × 320 pixels.

Simultaneously with the HSV recordings, time-synchronous acoustic and electroglottographic (EGG) signals were captured with a National Instruments (Austin, Texas, USA) DAC USB-6251 interface at a sampling rate of 20,000 Hz. The DAC was automatically triggered (i.e., switched on and off) via a TTL signal emitted by the HSV camera. The acoustic signal was captured with an omnidirectional microphone (Behringer ECM-8000, Behringer, or Sennheiser ME 62, Sennheiser, Wedemark, Germany) and a Mackie 802VLZ4 (Bothell WA, USA) preamplifier. The EGG signal was acquired using a dual-channel EGG device (EG2-PCX2, Glottal Enterprises, Syracuse, NY, USA). Due to the lack of a hardware low-pass filter and because of the relatively low sampling frequency of 20,000 Hz (with a Nyquist frequency of 10,000 Hz), the acoustic signals contained mild traces of aliasing artifacts. Spectrographic inspection of these acoustic signals using a spectrogram dynamic range of 90 dB revealed that the aliasing artifacts were on average above 5267 Hz, with a standard deviation of about 900 Hz. This suggests that—while the spectral acoustic data needs to be considered with care—it was safe to utilize the acoustic signals for both fundamental frequency estimation and computation of the intensity relation of the first to the second harmonic found in the signals. That latter approach is justified because even for the highest investigated musical pitch (G6) at about 1568 Hz, the second harmonic was well below the lowest frequency where aliasing artifacts were observed in the acoustic signals.

In order to verify the accuracy of the signals’ synchronization, a custom-made rotating disk with a printed black and white pattern was synchronously recorded with both HSV and a simple electric circuit containing a photodiode that monitored the rotating disk’s light intensity. The output of the photodiode current was routed to the acoustic channel of the DAC. The digitally computed light intensity of the respective HSV recording was compared with the photodiode current variation seen in the DAC’s input channel, and a perfect temporal agreement was found.

### HSV pre-processing and data analysis

The segmentation of the visible glottis and the medio-lateral vocal fold deflections—as documented by HSV—required several pre-processing steps that were accomplished through scripts implemented in the Matlab framework (R2014b, MathWorks Inc., Natick, MA, USA)^45,59–61^:

- In some cases, a honeycomb structure introduced by the endoscope optics was visible in the HSV recordings. This artifact was removed with a frequency-selective FFT-filter by transforming the images into the frequency domain via a 2-D discrete Fourier transform. Therein, the periodic noise appeared as 2 major peaks, apart from the center frequency peak. These two peaks were identified via an adaptable threshold binarization, and the areas were slightly increased via an opening filter and set to 0. The images were then transformed back into the image domain.
- Because the angle of the glottis could change with respect to the orientation of the HSV field of view during the recordings, the HSV footage had to be spatially rotated in order to align the glottis with the vertical dimension in the recordings. To this end, an approximate mask of the glottal opening was found in every image by means of time-difference images. In these ellipsoid-shaped masks, the orientation of the main axis of the glottis-opening could be calculated. Here, we assumed that the glottis shows the most movement between discrete images in the video. Hence, we calculated difference images from pictures with an adaptable time offset (typically set to 5 frames). Via various image processing methods, such as binarization, opening filter, and search for connected components, the largest object with the greatest movement was identified. The major axes of the resulting elliptical object were then determined, and the whole image could then be rotated so that the glottis was in vertical alignment. In most cases, some manual adjustments of the resulting angle graphs were necessary in order to remove sudden and improbable changes.
- In a last pre-processing step, a bounding box was manually drawn around the glottis at key frames, and all images of a sequence were correspondingly cropped. As a result, the glottis now appeared both vertically and horizontally centered in every frame of the HSV sequence.

Glottis segmentation—and thus determination of the time-varying medio-lateral vocal fold displacement along the antero-posterior glottal dimension—was performed using the custom-made Glottis Analysis Tools (Denis Dubrovskiy and Michael Döllinger, Erlangen University, Germany), as described previously^62^.

For further analysis, the glottal area waveform (GAW) was computed, producing the time-varying visible area of the glottis, indicated in pixels. The glottal segmentation data was also utilized to compute phonovibrograms^63^, i. e., a visualization procedure that extracts vocal fold vibrations from HSV data and transfers the motion information into a set of displacement data for both the left and the right vocal fold^63^. The PVG information pertaining to the individual vocal folds was then combined to a glottovibrogram^64^ (GVG), representing the time-varying glottal width in pixels along the antero-posterior glottal dimension. The GVG data was then used to compute the glottal closed quotients (i. e., the relative duration of vocal fold collision per vibratory cycle, expressed in percent) along the antero-posterior glottal axis. These last two processing steps, as well as the generation and assembly of all figures in this manuscript, were achieved with scripts written in the Python programming language.

### Analysis of voice signals

After recording and segmentation of the glottis from the HSV material all voice signals (GAW, EGG signal and the audio signal, respectively) were analyzed concerning *f*_*o*_ using an auto-correlation method within the custom made MultiSignalAnalyzer Software^65^. In order to avoid irregularities occurring during possible *f*_*o*_ transitions, only the stable part of each phonation was analyzed using a time window of 100 ms at the temporal midpoint of each phonatory (musical) pitch (midpoint ± 50 ms). The relative sound level difference between the first and the second harmonic (H1–H2, expressed in decibel (dB)^66^) was computed for all phonations at lowest and highest phonations, attempted at musical pitches C6 and G6. Because of some aliasing phenomena in the audio signals, calibration of the sound pressure level was considered problematic.

### Aeroacoustic model

For the aeroacoustic model used for computing the data shown in Fig. 2 we applied the following reasoning. When, in a hypothetical aeroacoustic sound source without vocal fold vibration, the glottal air flow separates from the glottis and a jet is formed, small instabilities in this glottal air jet can become entrained at certain frequencies due to a feedback loop between these downstream-traveling flow structures and acoustic waves traveling upstream^20^. For an impinging jet model, the emerging frequencies have been estimated with a previously^20^ described model established by *f*_*n*_ = *n* · *u*/*x*_*wall*_, where is the *f*_*n*_ frequency of the *n*^th^ possible whistle frequency, *n* is the mode number, *x*_*wall*_ is the jet length, and *u* is the mean convection speed of downstream moving coherent structures (i.e., the air flow speed). The mean convection *u* is approximated by *u* = *V*/*A*_*gl*_., where *V* is the volumetric air flow rate and *A* is the glottal constriction area. For the lowest possible frequency (mode-1), the model is reduced to *f*_*mode-1*_ = *u*/*x*_*wall*_.

### Computer simulation

A finite difference model of vocal fold tissue vibration was used to generate a high *f*_*o*_ with string-like restoring forces. However, a single string is insufficient to produce self-sustained oscillation with air passing over its surface. It requires multiple coupled strings with ribbon-like flexing of the medial surface of the vocal folds. Alternating convergent and divergent glottal shapes can then produce an aerodynamic push–pull on the vocal folds for sustained oscillation^67^. The vocal folds also have tissue depth in the medial–lateral direction that allows edge movement but restrains movement into the deep muscular layer. A rectangular parallelepiped with 90 coupled masses was sufficient to meet the boundary conditions and the tissue properties. The vocal fold length was 0.945 cm, the thickness 0.3 cm, and the depth 0.45 cm. In the anterior–posterior direction, 5 masses allowed string-like motion with fixed boundary conditions at both ends. Along the vocal fold thickness, 3 masses provided the ribbon-like flexure and bi-stable nature of voice registration^68^, and 6 masses were used laterally to allow vibration to dissipate to zero. The masses were coupled with fiber stresses of 0.9 MPa along the vocal fold length in the first two medial–lateral layers that represented the mucosa and ligament, respectively. In the 3—6 medial–lateral layers, a muscle fiber stress 5 kPa was selected, a value in the mid-range of measured thyroarytenoid muscle stress^69^. For shear coupling between the fibers, a gel shear modulus of 1.0 kPa was chosen according to measurement^70^. The damping ratio for the vibrating tissue needed to be 0.04, lower than the 0.1 value typically chosen for speech-like fundamental frequencies^71^. With this low damping ratio, it required a 4 kPa lung pressure to obtain self-sustained oscillation. For the 90 masses, 180 first-order differential equations were solved with a 4-th order Runge–Kutta solver^14^. With a simple string formula based on ligament stress and vocal fold length, the natural frequency of oscillation was predicted to be 1587 Hz.

Vocal fold adduction was controlled with one variable, the superior-posterior glottal width, which is the distance between the vocal processes of the arytenoid cartilages (i.e., the posterior cartilaginous boundary of the membranous vocal fold portion). This width was chosen to be 0.1 mm for tight adduction and 0.6 mm for weak adduction. The width varied linearly to zero at the anterior commissure. However, the glottal width did not vary vertically along the thickness of the vocal folds. In other words, the pre-phonatory glottis was neither convergent nor divergent, but rectangular.

The aero-acoustic solution was obtained for an [a:] vowel with a simplified Navier–Stokes approach as described in^68^. The airway geometry, from the trachea to the lips, was taken from MRI data obtained by Story et al.^72^.

### Ethical votum

Freiburg University 380/12.

### Supplementary Information

Supplementary Figures.

## Data Availability

The datasets generated during and/or analysed during the current study are available from the corresponding author on reasonable request.

## References

[1] Baken, R. J. & Orlikoff, R. F. Clinical Measurement of Speech and Voice. (Singular Thomson Learning, 2000).

[2] Herbst CT (2020). The snake pit of voice pedagogy part I: Proprioception, perception, and laryngeal mechanisms. J. Sing..

[3] Echternach M, Zehnhoff-Dinnesen A, Wiskirska-Woznika B, Neumann K, Nawka T (2020). Vocal registers. European Manual of Medicine.

[4] Walker JS (1988). An investigation of the whistle register in the female voice. J. Voice.

[5] Miller DG, Schutte HK (1993). Physical definition of the ‘flageolet register’. J. Voice.

[6] Keilmann A, Michek F (1993). Physiologie und akustische Analysen der Pfeifstimme der Frau. Folia Phoniatr. Et Logop.

[7] Kiesgen P (2006). Voice Pedagogy: Registration. J. Sing.

[8] Garnier M, Henrich N, Crevier-Buchman L, Vincent C, Smith J, Wolfe J (2012). Glottal behavior in the high soprano range and the transition to the whistle register. J. Acoust. Soc. Am..

[9] Titze I (1994). Principles of voice production.

[10] Henrich N (2006). Mirroring the voice from Garcia to the present day: Some insights into singing voice registers. Logop. Phoniatr. Vocology.

[11] Herbst CT (2021). 2021 Registers—The snake pit of voice pedagogy. Part 2: mixed voice, vocal tract influences, Individual Teaching Systems. J. Sing.

[12] Van den Berg J (1958). Myoelastic-aerodynamic theory of voice production. J. Speech Hear. Res..

[13] Herbst CT, Elemans CPH, Tokuda IT, Chatziioannou V, Švec JG (2023). Dynamic system coupling in voice production. J. Voice.

[14] Titze, I. R. *The Myoelastic Aerodynamic Theory of Phonation*. (The National Center for Voice and Speech, 2006). 10.1044/jshr.2303.495 7421153

[15] Švec JG, Schutte HK, Chen CJ, Titze IR (2023). integrative insights into the Myoelastic-Aerodynamic theory and acoustics of phonation. Scientific tribute to Donald G. Miller. J. Voice.

[16] Schoder S, Maurerlehner P, Wurzinger A, Hauser A, Falk S, Kniesburges S, Döllinger M, Kaltenbacher M (2021). Aeroacoustic sound source characterization of the human voice production—Perturbed convective wave equation. Appl. Sci..

[17] Fant G (1979). Glottal source and excitation analysis. STL-QPSR.

[18] Roberts LH (1975). The rodent ultrasound production mechanism. Ultrasonics.

[19] Mahrt E, Agarwal A, Perkel D, Portfors C, Elemans CPH (2016). Mice produce ultrasonic vocalizations by intra-laryngeal planar impinging jets. Curr. Biol..

[20] Håkansson J (2022). Aerodynamics and motor control of ultrasonic vocalizations for social communication in mice and rats. BMC Biol..

[21] Riede T, Borgard HL, Pasch B (2017). Laryngeal airway reconstruction indicates that rodent ultrasonic vocalizations are produced by an edge-tone mechanism. R Soc. Open Sci..

[22] Klatt D, Klatt L (1990). Analysis, synthesis, and perception of voice quality variations among female and male talkers. J. Acoust. Soc. Am..

[23] Harrison DFN (1995). The anatomy and physiology of the mammalian larynx.

[24] Schultz, P. Über einen Fall von willkürlichem laryngealen Pfeifen beim Menschen. *Arch f. Physiol.*, *Arch f. Physiol.* Suppl. 523, (1902).

[25] Berry DA, Herzel H, Titze IR, Story BH (1996). Bifurcations in excised larynx experiments. J. Voice.

[26] Tsai CG, Shau YW, Liu HM, Hsiao TY (2008). Laryngeal mechanisms during human 4-kHz vocalization studied with CT videostroboscopy and color Doppler imaging. J. Voice.

[27] Edgerton M, Howard DM, Nix J, Welch G (2013). The extra-normal voice. The Oxford Handbook of Singing.

[28] Corcia AD, Fussi F, Izdebski K (2016). Whistle register and M3: A preliminary HSDI investigation by visualization and acoustics in male and female singers. Normal and abnormal vocal folds kinematics: high speed digital phonoscopy (HSDP), optical coherence tomography (OCT) & narrow imaging.

[29] Herzel H, Reuter R (1997). Whistle register and biphonation in a child’s voice. Folia Phoniatr Logop.

[30] Döllinger M, Berry DA, Luegmair G, Hüttner B, Bohr C (2012). Effects of the Epilarynx area on vocal fold dynamics and the primary voice signal. J. Voice.

[31] Edgerton ME, Tan SW, Evans G, Myung HJ, Bo KK, Loo FY, Pan KC, Hashim MN (2013). Pitch profile of the glottal whistle (M4). Malays. J. Sci..

[32] Švec JG, Sundberg J, Hertegård S (2008). Three registers in an untrained female singer analyzed by videokymography, strobolaryngoscopy and sound spectrography. J. Acoust. Soc. Am..

[33] Echternach M, Döllinger M, Sundberg J, Traser L, Richter B (2013). Vocal fold vibrations at high soprano fundamental frequencies. J. Acoust. Soc. Am..

[34] Izdebski K, Di Lorenzo E, Yan Y, Blanco M (2016). What we have learned about ingressive phonation and whistle voice production from HSDP. Normal and Abnormal Vocal Folds Kinematics: High Speed Digital Phonoscopy (HSDP), Optical Coherence Tomography (OCT) & Narrow Imaging.

[35] Sakakibara K-I (2003). Production mechanism of voice quality in singing. J. Phon. Soc. Jpn..

[36] Lã FMB, Sundberg J, Granqvist S (2022). Augmented visual-feedback of airflow: Immediate effects on voice-source characteristics of students of singing. Psychol. Music.

[37] Miller, D. G. Registers in Singing. Empirical and Systematic Studies in the Theory of the Singing Voice. *Doctoral Dissertation* (University, 2000).

[38] Childers DG, Naik JM, Larar JN, Krishnamurthy AK, Moore GP, Titze IR, Scherer R (1983). Electroglottography, speech, and ultra-high speed cinematography. Vocal Fold Physiology and Biophysics of Voice 202–220.

[39] Lohscheller J, Švec JG, Döllinger M (2013). Vocal fold vibration amplitude, open quotient, speed quotient and their variability along glottal length: kymographic data from normal subjects. Logoped. Phoniatr. Vocol..

[40] Brudzynski SM, Kehoe P, Callahan M (1999). Sonographic structure of isolation-induced ultrasonic calls of rat pups. Dev Psychobiol.

[41] Shadle C (1983). Experiments on the acoustics of whistling. Phys. Teach..

[42] Titze I, Riede T, Mau T (2016). Predicting achievable fundamental frequency ranges in vocalization across species. PLoS Comput. Biol..

[43] Henrich N, d’Alessandro C, Doval B, Castellengo M (2005). Glottal open quotient in singing: Measurements and correlation with laryngeal mechanisms, vocal intensity, and fundamental frequency. J. Acoust. Soc. Am..

[44] Herbst CT, Schutte HK, Bowling DL, Svec JG (2017). Comparing chalk with cheese—The EGG contact quotient is only a limited surrogate of the closed quotient. J. Voice.

[45] Echternach M, Burk F, Koberlein M, Selamtzis A, Dollinger M, Burdumy M, Richter B, Herbst CT (2017). Laryngeal evidence for the first and second passaggio in professionally trained sopranos. PLoS One.

[46] Kato H, Lee Y, Wakamiya K, Nakagawa T, Kaburagi T (2023). Vocal fold vibration of the whistle register observed by high-speed digital imaging. J. Voice.

[47] Titze IR (2008). A hypothesis about whistle voice. J. Sing..

[48] Garnier M, Henrich N, Smith J, Wolfe J (2010). Vocal tract adjustments in the high soprano range. J. Acoust. Soc. Am..

[49] Echternach M (2015). Articulation and vocal tract acoustics at soprano subject’s high fundamental frequencies. J. Acoust. Soc. Am..

[50] Köberlein M (2021). Investigation of resonance strategies of high pitch singing sopranos using dynamic three-dimensional magnetic resonance imaging. J. Acoust. Soc. Am..

[51] Pressman JJ, Kelemen G (1955). Physiology of the larynx. Physiol. Rev..

[52] Van den Berg, J. Vocal ligaments versus registers. *The NATS Bulletin* 16–31 (1963).

[53] Titze IR, Hunter EJ (2004). Normal vibration frequencies of the vocal ligament. J. Acoust. Soc. Am..

[54] Thurman, L., Welch, G. F., Theimer, A. & Klitzke, C. Addressing Vocal Register Discrepancies: An Alternative, Science-Based Theory Of Register Phenomena. in *Second International Conference The Physiology and Acoustics of Singing (PAS2)* 64 (National Center for Voice and Speech, 2004).

[55] Baken RJ, Isshiki N (1977). Arytenoid displacement by simulated intrinsic muscle contraction. Folia Phoniatr (Basel).

[56] Edgerton ME (2015). The 21st-century voice : Contemporary and traditional extra-normal voice.

[57] Bunch M, Chapman J (2000). Taxonomy of singers used as subjects in scientific research. J. Voice.

[58] Joliveau E, Smith J, Wolfe J (2004). Tuning of vocal tract resonance by sopranos. Nature.

[59] Echternach M, Burk F, Köberlein M, Burdumy M, Döllinger M, Richter.  (2017). The influence of vowels on vocal fold dynamics in the tenor's passaggio. J. Voice.

[60] Echternach M, Döllinger M, Köberlein M, Kuranova L, Kainz MA (2021). Influence of loudness on vocal stability in the male Passaggio. J. Voice.

[61] Echternach M, Herbst CT, Köberlein M, Story B, Döllinger M, Gellrich D (2021). Are source-filter interactions detectable in classical singing during vowel glides?. J. Acoust. Soc. Am..

[62] Inwald EC, Döllinger M, Schuster M, Eysholdt U, Bohr C (2011). Multiparametric analysis of vocal fold vibrations in healthy and disordered voices in high-speed imaging. J. Voice.

[63] Lohscheller J, Eysholdt U (2008). Phonovibrogram visualization of entire vocal fold dynamics. Laryngoscope.

[64] Karakozoglou S-Z, Henrich N, d’Alessandro C, Stylianou Y (2012). Automatic glottal segmentation using local-based active contours and application to glottovibrography. Speech Commun..

[65] Schäffner FD (2015). Signalerfassung und Analyse medizinischer Daten: Entwicklung einer Analysesoftware für medizinische Messdaten innerhalb der Stimmforschung.

[66] Sundberg J, Andersson M, Hultqvist C (1999). Effects of subglottal pressure variation on professional baritone singers’ voice sources. J. Acoust. Soc. Am..

[67] Titze IR (1988). The physics of small-amplitude oscillation of the vocal folds. J. Acoust. Soc. Am..

[68] Titze IR (2014). Bi-stable vocal fold adduction: A mechanism of modal-falsetto register shifts and mixed registration. J. Acoust. Soc. Am..

[69] Alipour-Haghighi F, Titze IR, Perlman AL (1989). Tetanic contraction in vocal fold muscle. J. Speech Hear. Res..

[70] Chan RW, Titze IR (2000). Viscoelastic shear properties of human vocal fold mucosa: Theoretical characterization based on constitutive modeling. J. Acoust. Soc. Am..

[71] Ishizaka K, Flanagan JL (1972). Synthesis of voiced sounds from a two-mass model of the vocal cords. Bell Syst. Tech. J..

[72] Story BH, Titze IR (1998). Parametrization of vocal tract areafunctions by empirical orthogonal modes. J. Phonetics.

