# Supplementary material for: Biomechanics of sound production in high-pitched classical singing
Source: Sci Rep. 2024 Jun 7;14:13132. doi: 10.1038/s41598-024-62598-8 (PMC11161605; doi:10.1038/s41598-024-62598-8)
Supplement: Supplementary file 1 — Supplementary Figures. [file 41598_2024_62598_MOESM1_ESM.pdf]

Supplementary Materials

Title: Biomechanics of sound production in high-pitched classical singing

Echternach, M., Burk, F., Köberlein, M., Döllinger, M., Burdumy, M., Richter, B., Titze, I. R., Elemans, C. P. H., Herbst, C. T.

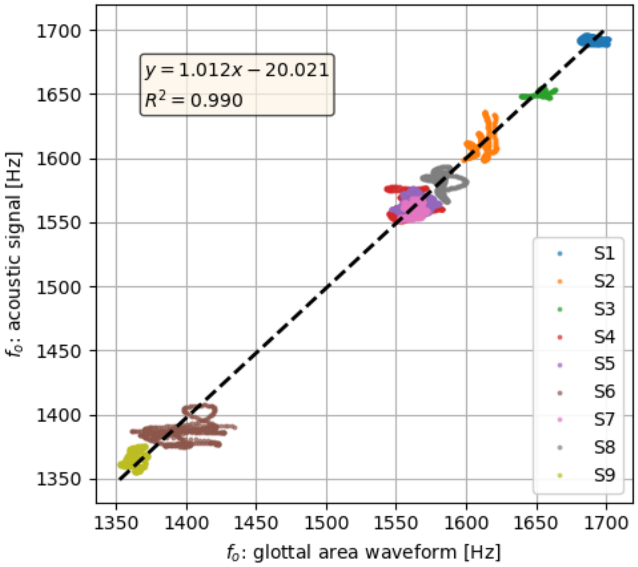

Figure S1. Correlation of f_o from glottal area waveform (GAW; abscissa) and acoustic signal (ordinate) for all target notes of all participants. There was an almost perfect correlation between the two data sets (R^2 =.99). Note that the slight discrepancy between the two data sets (GAW and acoustic) is caused by the time-delay of the acoustic signal with respect to the GAW signal: While the GAW signal is quasi-instantaneous, the acoustic signal is delayed by $\Delta t = d \times c$, c ≈ 340 ms. Unfortunately, the microphone distance from the glottis could not be reconstructed from the recording setup. For this reason, no time-correction of the acoustic signal could be performed.

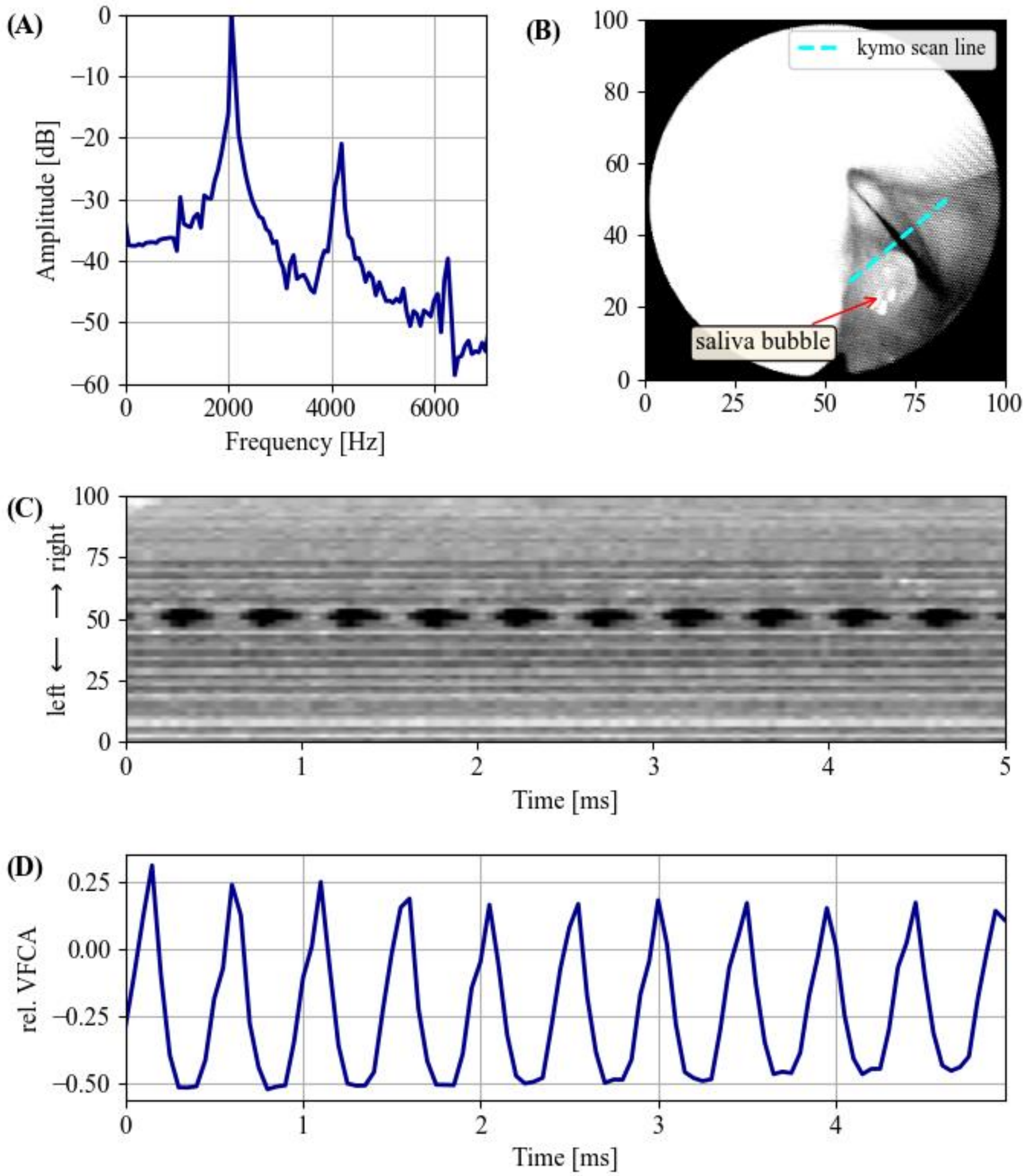

Figure S2. Documentation of phonation at pitch B6 (f_o ≈ 1975 Hz) by participant S3. (A) acoustic spectrum; (B) still image of HSV footage, captured at the instant of maximum glottal opening – the dashed cyan line indicates the videokymographic (VKG) scan line used for panel C; (C) VKG shows 10 glottal cycles; (D) glottal area waveform (GAW), documenting full glottal closure during each oscillatory cycle.

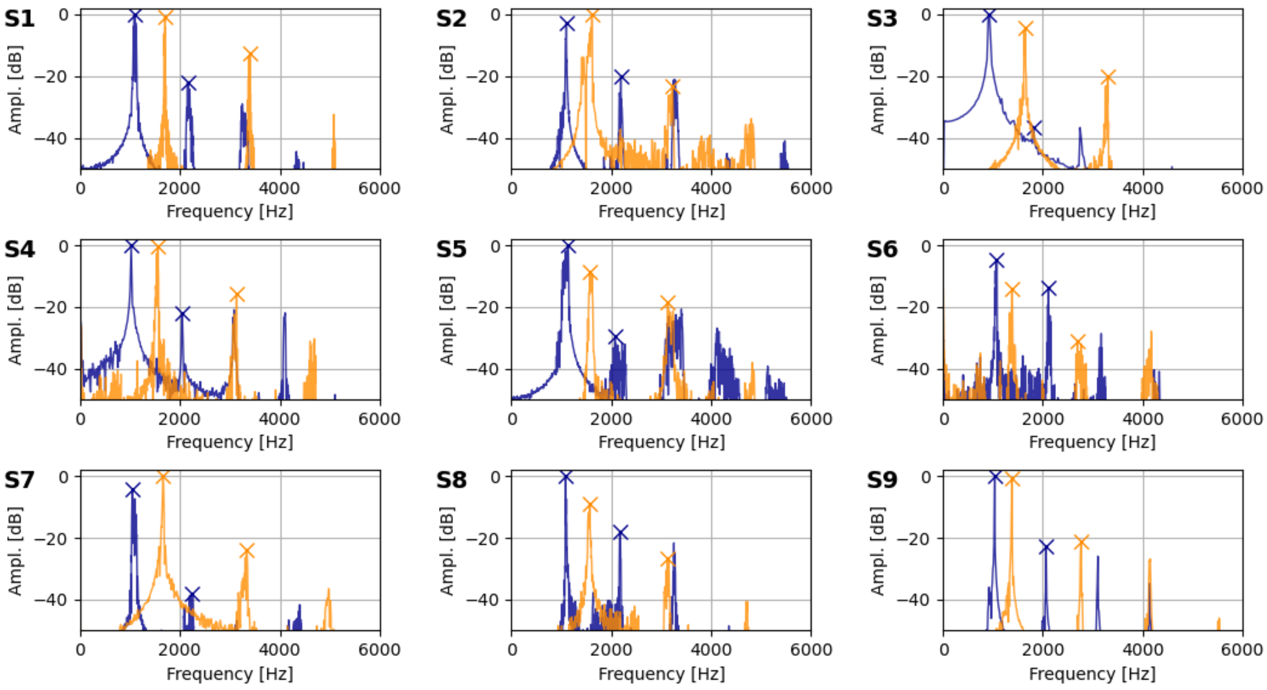

Figure S3. Spectral analysis of acoustic data captured from all nine participants, considering the lowest (blue) and highest (orange) respective phonations.

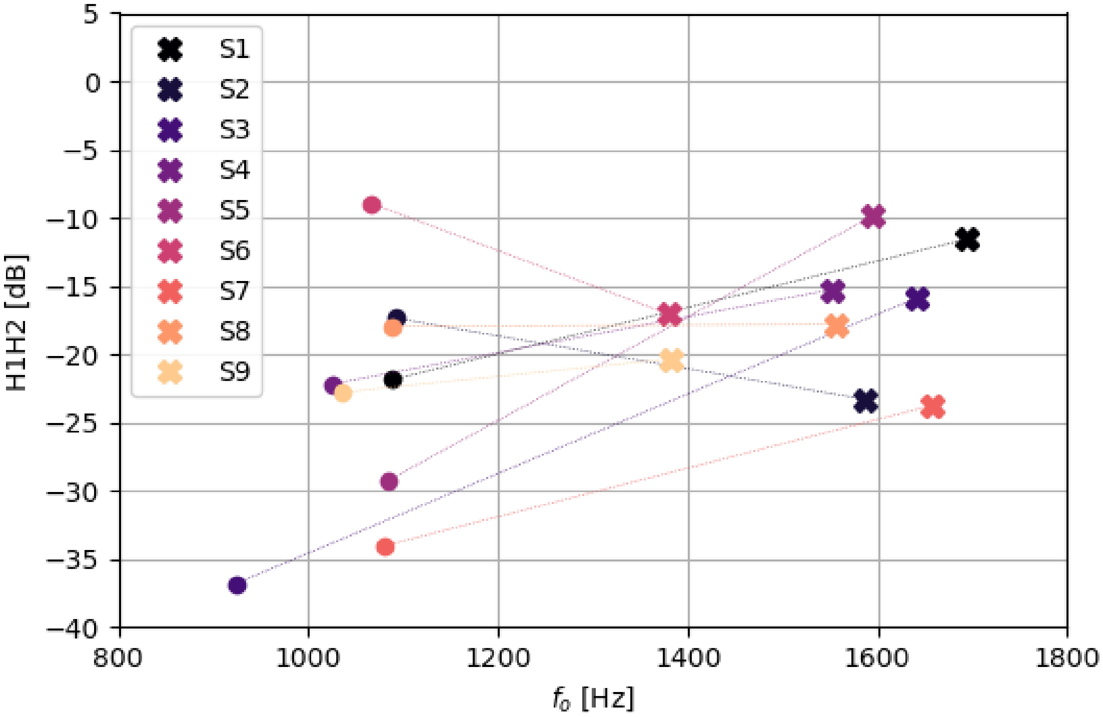

Figure S4. Relative sound level differences of the first versus the second harmonic of the acoustic signals (H1H2) as a function of phonatory fundamental frequency (f_o), shown for the respective lowest and highest phonations of all nine participants.

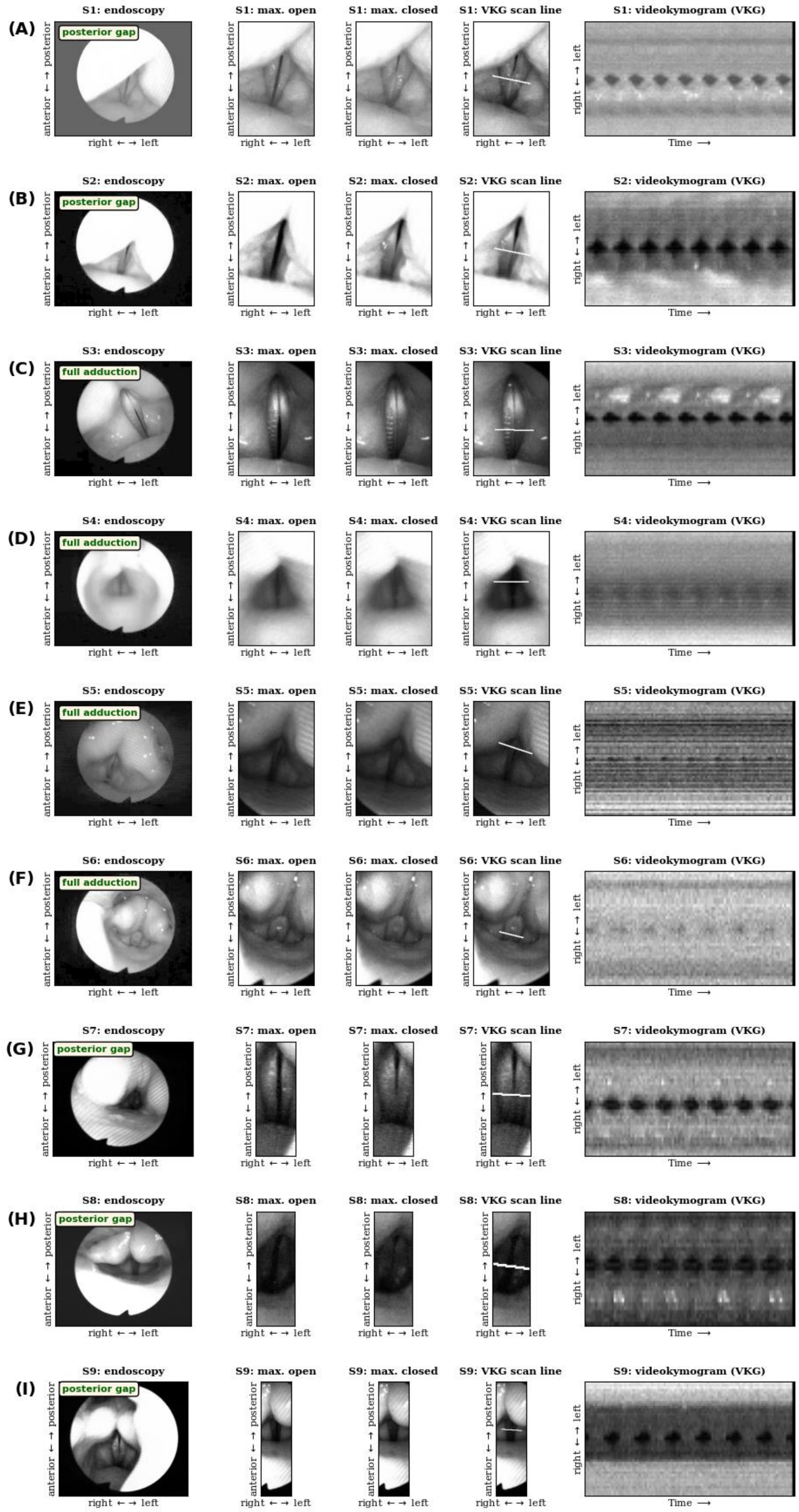

Figure S5. Documentation of vocal fold oscillation and glottal configuration of all participants when phonating at target notes (see column 4 in Table 1). Please refer to legend of Figure 3 for details.

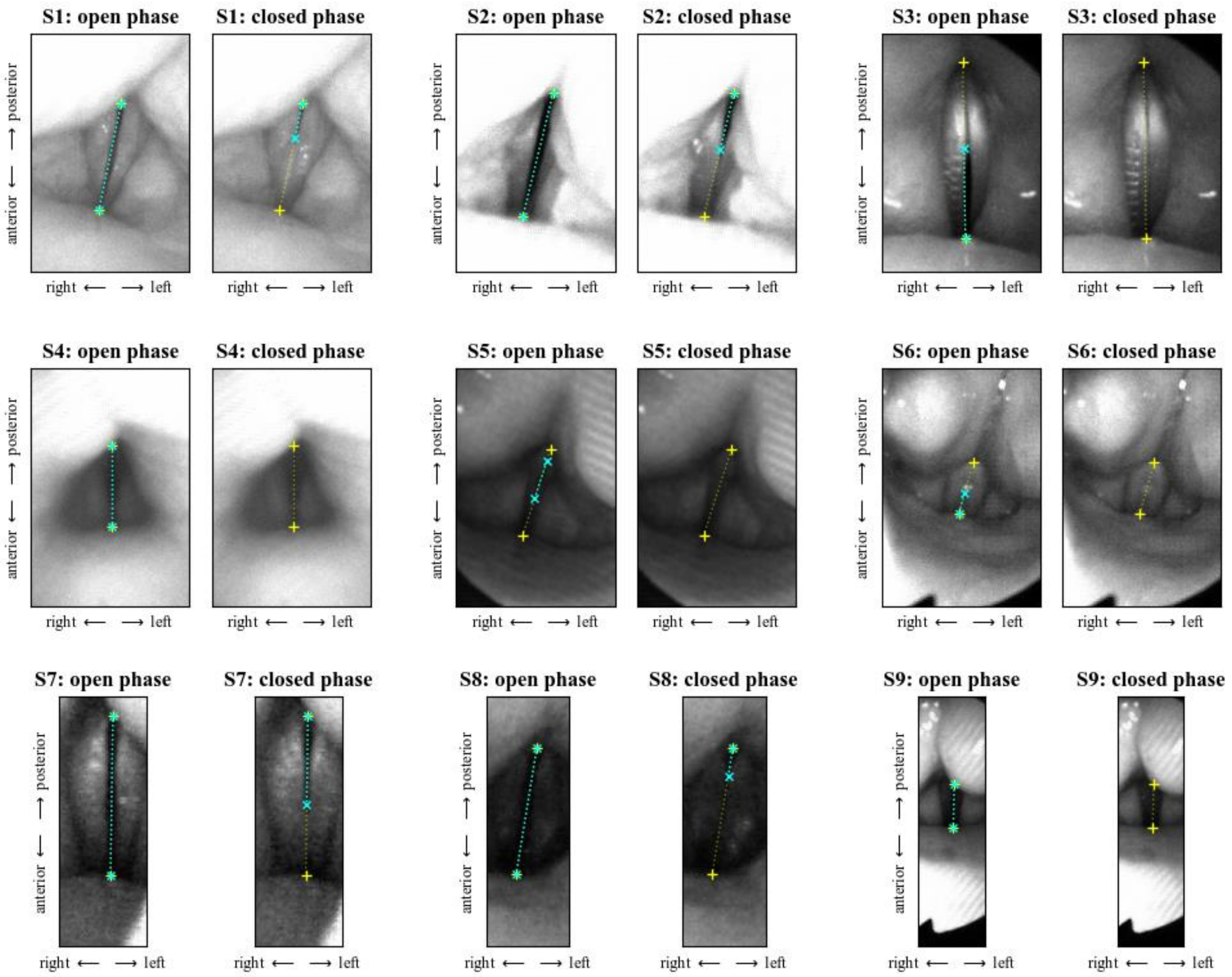

Figure S6. Glottal configurations found at target note phonation (see column 4 in Table 1) for all nine participants in both the open and the closed phase of vocal fold oscillation. The dashed yellow line and the + markers indicate the visible antero-posterior extension of the glottis. The dashed cyan line and the * and x markers indicate the observed glottal opening.
